# The Significance of Low Magnesium Levels in COVID-19 Patients

**DOI:** 10.3390/medicina59020279

**Published:** 2023-01-31

**Authors:** Adorata Elena Coman, Alexandr Ceasovschih, Antoneta Dacia Petroaie, Elena Popa, Cătălina Lionte, Cristina Bologa, Raluca Ecaterina Haliga, Adriana Cosmescu, Ana Maria Slănină, Agnes Iacinta Bacușcă, Victorița Șorodoc, Laurențiu Șorodoc

**Affiliations:** 1 Preventive Medicine and Interdisciplinarity Department, Grigore T. Popa University of Medicine and Pharmacy Iasi, 700115 Iasi, Romania; 2 2nd Internal Medicine Department, Sf. Spiridon Clinical Emergency Hospital, 700111 Iasi, Romania; 3 Internal Medicine Department, Faculty of Medicine, Grigore T. Popa University of Medicine and Pharmacy, 700115 Iasi, Romania

**Keywords:** COVID-19, SARS-CoV-2, magnesium, endothelial dysfunction, coagulation, inflammation, “long COVID-19” syndrome, pregnancy

## Abstract

Magnesium is the fourth most common mineral in the human body and the second richest intracellular cation. This element is necessary for many physiological reactions, especially in the cardiovascular and respiratory systems. COVID-19 is an infectious disease caused by SARS-CoV-2. The majority of people who become ill as a result of COVID-19 have mild-to-moderate symptoms and recover without specific treatment. Moreover, there are people who develop severe forms of COVID-19, which require highly specialized medical assistance. Magnesium deficiency may play a role in the pathophysiology of infection with SARS-CoV-2. The primary manifestation of COVID-19 remains respiratory, but the virus can spread to other organs and tissues, complicating the clinical picture and culminating in multiorgan failure. The key mechanisms involved in the disease include direct viral cytotoxicity, endothelial dysfunction, and exaggerated release of inflammatory cytokines. The aim of this review was to summarize the available data regarding the role of magnesium in COVID-19 patients and its particularities in different clinical settings.

## 1. Introduction

### 1.1. Coronavirus Disease 2019 (COVID-19) 

A newly discovered human coronavirus with high pathogenicity and an RNA envelope—severe acute respiratory syndrome coronavirus 2 (SARS-CoV-2)—led to coronavirus disease 2019 (COVID-19) and caused the worst pandemic of the last century [1]. Its global spread and considerable mortality and morbidity made it a major public health concern [2]. The outbreak started in December 2019 when atypical cases of viral pneumonia emerged in Wuhan, Hubei Province, China [2], which were epidemiologically linked to the Huanan Seafood Wholesale Market. Over 624 million confirmed COVID-19 cases and 6.5 million deaths have been recorded worldwide across 185 countries [3,4]. 

Coronaviruses are enveloped, positive, single-stranded, large, ribonucleic acid spherical viruses with a core shell and characteristic “crown-like” spikes on their surfaces [5]. They are capable of causing infection in humans and animals. Divided into four subsets (alpha-, beta-, gamma-, and deltacoronaviruses), the novel coronavirus belongs to the beta lineage, a subgroup with origins in bats (96% identical at the whole-genome level) [6] that has potentially fatal outcomes [2]. Genetic reports highlight the distinct traits of SARS-CoV-2, e.g., superior linkage to the angiotensin-converting enzyme 2 (ACE-2) receptor, which is central to the virus’s pathogenesis, and a polybasic cleavage site at the S1/S2 spike junction, which determines the host range and virulence [2]. Virions attack human cells via attaching spike proteins to the ACE-2 receptors of organism cells [7]. 

Regarding its transmission, the central pathway is the respiratory tract. In addition, droplet transmission, aerosol, saliva, eye, urine, stool, and inanimate surface transmission are recognized as possible routes [2], while no vertical transmission has been noticed [5]. An average of 80% of COVID-19 infections are mild. In addition, 15% of cases that present as serious forms of the disorder require hospital care, with 5% of these requiring intensive care. From 2% to 5% of cases result in mortality, with this percentage being higher in victims requiring invasive mechanical ventilation. The main cause of death in COVID-19 patients is documented as acute respiratory distress. The cardiovascular system and shock are also often implicated [5,8]. 

Firstly, COVID-19 affects the cardiovascular system through contact with the myocard and endothelium via (ACE-2) receptor-mediated endocytosis, which is classified as respiratory system damage [1]. Then, the virus replicates, protein synthesis occurs, and copies are made for transduction [7]. Among cardiac-resident cells, e.g., endothelial cells, pericytes, and cardiomyocytes, ACE-2 is the principal enzyme in the local clearance of Ang II [9]. It was reported that approximately 12% of COVID-19 patients suffer cardiac injury [10]. The cardiovascular system is usually altered, with elevation of cardiac biomarkers, myocardial injury, myocarditis, acute myocardial infarction, heart failure, dysrhythmias, venous and arterial thromboembolic events, and cardiogenic shock and arrest being observed [2,11]. Middle-aged males and elderly patients (probably due to endothelial dysfunction and the loss of endogenous cardioprotective mechanisms) [9] with severe medical statuses are the most vulnerable in this regard. 

The synergy between SARS-CoV-2 and ACE-2 receptors (signaled on pneumocytes and endothelial cells) via the S-spike protein transforms the ACE-2 pathways, causing acute injury to the lungs, heart, and endothelial cells [2]. Vascular smooth muscle cells have both ACE-2 receptors and TMPRSS2 protease on their cell membranes, allowing the virus direct entry. The downregulation of ACE-2 triggers the expression of oligopeptide angiotensin Ang II. ACE-2 depletion increases neutrophil infiltration in the infarct area. Therefore, the activation of the molecular pathway cascade enables the immune reply [7]. Proinflammatory cytokines, including interleukin (IL)-2, IL-10, IL-6, and IL-8, as well as tumor necrosis factor (TNF)-α, injure cardiac myocytes and the vascular endothelium in both phases (infection with the virus and extreme inflammation), ending in acute respiratory distress syndrome (ARDS) and other end-organ injury [2]. Cytokine release promotes leukocyte adhesion molecule expression on endothelial cells covering previous atheroma, stimulating the local recruitment of these inflammatory cells [10]. Signs of early SARS-CoV-2 infection are distal vasculitis with acrosyndrome and dyshydrosis in the terminal digits [7]. 

Thus, SARS-CoV-2 may exert direct cardiotoxicity resulting in viral myocarditis. In the majority of cases, myocardial damage presents as an excessive cardiometabolic burden combined with a systemic infection and ongoing hypoxia due to severe pneumonia or ARDS. Plaque disruption and acute coronary syndrome emerge from systemic inflammation, which increases vascular shear stress [10,12] and catecholamine surges or coronary thrombosis in COVID-19 patients [2]. Among nonischemic mechanisms, acute and fulminant myocarditis and stress-induced cardiomyopathy were reported [4].

Other systemic COVID-19 disease outcomes, such as sepsis and disseminated intravascular coagulation (DIC), also contribute to cardiac damage. These mechanisms are represented by morphopathological changes in the form of the cardiac tissue, interstitial inflammatory infiltration, and myocyte necrosis. Furthermore, endothelial vascular inflammation and microthrombosis may occur [2].

Consequently, the aforementioned mechanisms involved in multiple-organ damage determine the severe evolution and unfavorable prognosis of patients with COVID-19.

### 1.2. Magnesium

Magnesium is the fourth most common mineral in the human body (after calcium, potassium, and sodium) and the second intracellular cation, the richest after potassium [13]. This element is necessary for many physiological reactions, especially in the cardiovascular and respiratory systems [14]. For normal cell membrane function, the activation of adenosine triphosphatase (ATPase) by magnesium is essential, and this activation is the energy source Na^+^-K^+^ pumping. Increases in intracellular Na^+^ and Ca^2+^ and a loss in K^+^ can be determined by intracellular magnesium deficiency [15]. 

Regarding the distribution of magnesium in the body, approximately 99% of the total body magnesium is found in the bones, muscles, and nonmuscular soft tissue. In addition, 1% of the total body magnesium is located in red blood cells and serum (extracellular magnesium) and can be categorized into three classes: free, or ionized; bound to protein; or complexed with anions, such as phosphate, bicarbonate, and citrate or sulphate. Moreover, 1–5% of intracellular magnesium is ionized (from a total of 5 to 20 mmol/L), with the remainder being bound to proteins, negatively charged molecules, and adenosine triphosphate [16].

Magnesium acts as a cofactor of numerous enzymes, regulating ion channels and energy generation, and it is essential for maintaining normal cellular physiology and metabolism. Among the roles magnesium plays, function of the heart, modulation of neuronal excitation, intracardiac conduction, and myocardial contraction via the regulation of several ion transporters, including the potassium and calcium channels, are of importance. In addition, it has a significant role in regulating vascular tone, atherogenesis, thrombosis, and vascular calcification, as well as the proliferation and migration of endothelial and vascular smooth muscle cells. The risk of cardiovascular disease can be increased by kidney diseases, as they can lead to magnesium reserve depletion or overload, as the major magnesium homeostasis regulator is the kidney. It is important that magnesium supplementation follows biological evidence of hypomagnesemia and the existence of a causal link between this and heart disease, as there have been numerous observational studies that have demonstrated an association between low serum magnesium concentration or magnesium intake and increased atherosclerosis, coronary heart disease, arrhythmias, and heart failure prevalence [13,17,18].

Since magnesium is a ubiquitous element, several studies have been conducted on the role of serum and dietary magnesium intake in cardiovascular diseases, carotid intima-media thickness, arterial hypertension, and cholesterol synthesis. Various observational and interventional studies have shown that a low level of serum magnesium is associated with several cardiovascular risk factors and with a greater carotid intima-media thickness. Magnesium administration can prevent cardiovascular diseases, and it is a neuroprotective agent [19,20,21,22,23,24]. Moreover, low magnesium levels are implicated in endothelial dysfunction and inflammation, resulting in increased C-reactive protein (CRP) and cytokine secretion, increased nuclear factor kappa B (NF-κB), and platelet dysfunction, which can lead to thrombosis. An optimal dose of magnesium has not been defined, although it has a vital function in stability and cardiovascular health; however, the combination of magnesium and statin has great potential to reduce cholesterol, CRP, and carotid intima-media thickness. The timely use of this combination can reduce the morbidity and mortality associated with strokes [25,26,27,28].

A functional endothelium modulates the state of contraction of the underlying vascular smooth muscle and synthesizes the prostacyclin and nitric oxide vasodilators. In a state of endothelial dysfunction, it is involved in several disease processes, including atherosclerosis, pathological coronary vasoreactivity with or without significant coronary stenosis, and preeclampsia-eclampsia. Prostacyclin endothelial release was increased by magnesium infusion in cultured human endothelial cells in healthy, nonpregnant volunteers and preeclamptic patients, which suggested that the actions of vascular magnesium are mediated by prostacyclin release. In preeclampsia, pathologic platelet adhesion, aggregation, and the resulting microvascular occlusion secondary to endothelial dysfunction are antagonized by the release of prostacyclin, which is induced by magnesium [29,30].

### 1.3. Magnesium and Endothelial Dysfunction

The vascular endothelium has a major role in supporting vascular homeostasis through local modulators. These actively and reactively control the vascular tone, cell adhesion, vascular inflammation, and smooth muscle proliferation process [31]. 

Since it disrupts the homeostasis of endothelial cells, magnesium deficiency, combined with cardiovascular risk factors, is considered a promoter of endothelial dysfunction. The impact of hypomagnesemia on endothelial functions is significant, as it contributes to the triggering of oxidative stress and the stimulation of endothelial cell oxidant production [32,33,34]. Contrarily, magnesium suppresses vascular oxidative stress [35]. An imbalance between the activity of endogenous pro-oxidative enzymes, including NADPH oxidase, xanthine oxidase, and the mitochondrial respiratory and antioxidant enzymes, such as superoxide dismutase, glutathione peroxidase, and catalase, results in oxidative stress [36]. 

The mechanisms by which the vascular endothelium reacts in this context are not yet clear, as the changes are complex and multifactorial [37]. The dual role of reactive oxygen species (ROS) is being increasingly discussed. Under basal conditions, ROS, such as superoxide anion (O_2_¯), hydroxyl radical (OH), and hydrogen peroxide (H_2_O_2_), may have an important role in intracellular signaling in the vascular endothelium. On the other hand, in the presence of excessive oxidative stress, ROS contribute to the onset of endothelial dysfunction by stimulating the prothrombotic and proinflammatory pathways in the vascular endothelium. Magnesium reduces the vulnerability of the vascular endothelium to free radicals [38,39]. 

Reactive oxygen species, particularly superoxide anion, mostly come from the membrane-associated nicotinamide dinucleotide (phosphate) (NADH/NADPH) oxidase enzyme complex and nonenzymatic sources. These pro-oxidative enzymes are functional in vascular endothelial and smooth muscle cell membranes, as they are important reservoirs of superoxide anion, an inhibitor of nitric oxide bioavailability. NADH/NADPH oxidase is stimulated by the vasoactive agonist angiotensin II, thrombin, tumor necrosis factor-α, and platelet-derived growth factor [40]. Certain studies demonstrated that endothelial cells grown in a low-magnesium culture medium induced NADPH oxidase activity in endothelial cells [41]. 

In parallel, magnesium deficiency reduces the activity of antioxidant enzymes, such as superoxide dismutase, glutathione peroxidase, and catalase, decreasing cell and tissue antioxidant concentrations and increasing the production of hydrogen peroxide and superoxide anion by inflammatory cells [42]. 

A specific characteristic of endothelial dysfunction is the impairment of endothelium-derived nitric oxide (EDNO) bioactivity, which is an efficient vasodilator and an antiproliferative molecule, through its action on smooth muscle cells. EDNO is produced from the conversion of L-arginine to L-citrulline in the presence of endothelial nitric oxide synthase. Because of oxidative stress, hypomagnesemia intervenes in the uncoupling of endothelial nitric oxide synthase. As a consequence, the formation of excess superoxide anions against nitric oxide is stimulated, leading to endothelial dysfunction [31]. Therefore, a reduction in nitric oxide synthesis or even its inactivation is followed by endothelial dysfunction. 

Extracellular hypomagnesemia increases endothelial permeability [41]. Physiologically, the endothelium works as a selectively permeable membrane. To preserve the barrier function, maintaining the structural and functional integrity of the endothelium is essential. The increased production of free radicals by endothelial cells or other cell types causes a significant increase in the permeability of the endothelial monolayer to different molecules that would not normally cross the endothelial barrier [37]. 

Various studies have suggested that TRPM7 and MagT1 are the specific transporters involved in magnesium homeostasis, favoring the entry of extracellular magnesium into the cell and regulating magnesium signaling [35,43]. Moreover, as a result of these transporters, magnesium regulates endothelial survival, proliferation, and motility. Magnesium controls the endothelial barrier function through the regulation of barrier-stabilizing mediators. Cytoskeletal reorganization and the expression of junction proteins, the key elements in the endothelium, are stimulated by magnesium treatment, while MagT1 or TRPM7 suppression induces opposing effects. It is evident that hypomagnesemia causes harmful effects on the integrity of the endothelium, and magnesium supplements may be effective in preventing or treating vascular dysfunction [35]. 

Magnesium, perceived as a calcium antagonist, reduces it signaling in the endothelium by competitively binding to the calcium channel. Similarly, magnesium competes for binding sites with sodium ions on cells. As a result of these actions, intracellular calcium and sodium decrease, the level of prostaglandin E increases, endothelial dysfunction improves, and endothelial vasodilation is triggered. Magnesium also intervenes in the intracellular regulation of K, controlling its transport through sodium-potassium pumping (Na^+^/K^+^-ATPase). Hyperpolarization of the vascular smooth muscle cells by stimulating Na^+^/K^+^-ATPase produces a vasodilator effect [35]. 

Data from the literature have shown significant improvements in arterial endothelial function and exercise duration in patients with coronary artery disease who receive oral magnesium therapy, as endothelial function is directly related to magnesium levels [44,45]. A similar study conducted on patients with type-2 diabetes demonstrated that choline and magnesium coadministration was more efficient in reducing inflammatory response and endothelial dysfunction than the administrations of choline or magnesium only [46]. There is a substantial relationship between macrovascular endothelial function and serum magnesium levels, as seen in patients with end-stage renal disease who benefit from hemodialysis. In contrast, microvascular endothelial function is not related to serum magnesium concentration [47].

### 1.4. Magnesium and Inflammation

Experimental studies performed in vivo and in vitro have demonstrated that magnesium modulates the inflammatory response. The decrease in extracellular magnesium activated the transcription of NFkB in the endothelium cells [41]. NFkB has a role in triggering the global immune and inflammatory responses and controlling the gene expressions of cytokines, chemokines, growth factors, and adhesion molecules. In conjugation with the inhibitory protein IkB, NFkB remains inactive in the cytoplasm. Through exposure to bacteria, viruses, cytokines, or oxidative stress, NFkB activation is initiated. In this stage, through the proteolysis of IkB, a nuclear recognition site is revealed, and NFkB translocates into the nucleus. It attaches to DNA and, thus, determines mRNA expression [48]. 

The consequence of NFkB activation is endothelial dysfunction, which triggers a proinflammatory and proatherogenic phenotype. It is certain that oxidative stress and chronic inflammation are inseparable phenomena. Nitric oxide produced by endothelial cells stabilizes IkB by inhibiting the expression of the adhesion molecule, clearly reducing the inflammatory response. Magnesium supplementation significantly attenuates the translocation of NFkB from the cytoplasm to the nucleus, inhibits the degradation of IkB in endothelial cells, and implicitly reduces the inflammatory response [49]. 

The central role in mediating the inflammatory response induced by magnesium deficiency is assigned to IL-1α, which produces chemokines and adhesion molecules by activating NFkB. These principally increase IL-8 and RANTES (regulated upon activation, normal T-cell expressed and secreted), which are chemokines that are overexpressed in patients with atherosclerotic lesions. Moreover, the inhibition of IL-1α prevents low-magnesium-induced adhesion of monocytoid cells to the endothelium [41]. 

Another important factor involved in inflammation seems to be substance P (SP), a peptide of the tachykinin family found in both the central and peripheral nervous systems [50]. Magnesium deficiency is associated with neurogenic inflammation mediated by the release of SP, a physiopathological event preceded by significant increases in inflammation parameters (circulating IL-1, IL-6, tumor necrosis factor TNFα, histamine, PGE2, white blood cells, and cardiac tissue inflammation) and in oxidative stress factors. By restricting magnesium, the inhibition of neutral endopeptidase (NEP), a specific SP-degrading enzyme, maintains a high level of neurogenic inflammation, leading to increased intestinal and cardiac dysfunction [51]. 

The mechanism by which magnesium deficiency produces inflammatory stress is closely related to the role of magnesium as an antagonist of calcium. Hypomagnesemia causes an increase in intracellular calcium by activating L-type calcium channels or by releasing it from intracellular stores, such as the sarcoplasmic reticulum. The release of TNFα is induced by the increase in intracellular calcium and, thus, the inflammatory response is initiated, resulting in the production of cytokines [52]. 

Decreases in extracellular magnesium concentration in experimental animals induced an inflammatory response that determined the stimulation of phagocytic cells, with increases in polymorphonuclear leukocytes, mainly neutrophils and eosinophils, and macrophages also noted. Moreover, an increase in proinflammatory cytokines, especially IL-6 and TNFα, was observed [53]. The production of these cytokines was significantly reduced by the increase in intracellular magnesium following a magnesium treatment in vivo [54]. The acute phase proteins of alpha2-macroglobulin and alpha1-acid glycoprotein, increased in parallel with the mRNA level that encoded them [55]. Magnesium is involved in the prevention of cardiovascular disease, diabetes, and metabolic syndrome by reducing systemic inflammation and improving endothelial dysfunction. In chronic diseases associated with magnesium deficiency, the most commonly used inflammatory marker is CRP. In the majority of cases, magnesium deficiency is associated with a low degree of inflammation or pathological conditions for which inflammatory stress is considered a risk factor. At normal serum magnesium concentrations, there is no significant improvement in inflammatory markers, probably due to other nutritional and metabolic factors affecting inflammatory and oxidative stress [35,52]. 

Magnesium deficiency has been shown to be accompanied by high CRP in individuals whose magnesium dietary intakes are below the RDA. Magnesium supplementation has significantly improved serum CRP levels [56,57]. Similar studies showed that the serum CRP level was elevated 1.94 times (*p* < 0.05) in children consuming less than 75% of their magnesium RDA [56]. The results from various meta-analyses, systematic reviews, and studies have shown that dietary magnesium intake is significantly and inversely associated with serum CRP levels [52,54,58,59,60,61]. An inverse association between magnesium intake and metabolic syndrome has also been reported [62,63]. The favorable impact of magnesium on systemic inflammation is also reflected in patients with diabetes. Regarding the relationship between magnesium intake and serum inflammatory marker levels and HOMA-IR, it seems that magnesium intake is significantly inversely related to hs-CRP, IL-6, fibrinogen, and HOMA-IR, and serum magnesium level is inversely related to hs-CRP and HOMA-IR [64]. Another meta-analysis concluded that there is an inverse relationship between dietary magnesium intake and serum magnesium concentrations with the risk of total cardiovascular events [65]. It is abundantly clear that magnesium deficiency maintains both hyperinflammation in acute inflammatory processes and low-grade inflammation in chronic diseases [66,67].

### 1.5. Magnesium and Coagulation Disturbances 

Magnesium is known to have antiplatelet and antithrombotic effects. One of the mechanisms by which magnesium decreases platelet activation is by inhibiting the production of thromboxane A2 (TXA2), the platelet-stimulating factor, in parallel with increasing the release of prostacyclin (PGI2), the platelet-inhibiting factor [68]. 

The role of the GPIIb/IIIa receptor from the platelet surface in the mechanism of platelet aggregation is significant. It is known that magnesium competes with calcium ions for the binding sites in the glycoprotein (Gp)IIb subunit and can inhibit the binding of fibrinogen to the GPIIb/IIIa complex by changing the conformation of the receptor. Thus, the interaction of platelets with fibrinogen no longer occurs, and platelet aggregation is suppressed [68]. 

Magnesium has an antithrombotic effect since it intervenes in the regulation of endothelial nitric oxide synthase, increasing the production of EDNO. By reducing the release of EDNO, low levels of magnesium induce vasoconstriction, increase the proliferation of endothelial cells, and stimulate the adhesion of platelets and inflammatory cells. Thus, favorable conditions are created for the appearance of a prothrombotic and proatherogenic endothelium [31]. 

TF is a transmembrane glycoprotein that functions as a receptor and cofactor for factor (F) VII/VIIa [69]. As a promoter of the coagulation cascade, the TF-FVIIa complex plays a crucial role in hemostasis. Factors IX–IXa and X–Xa are proteolytically activated by the TF-FVIIa complex. By activating the prothrombinase complex, thrombin is generated, resulting in fibrin formation and platelet activation. TF forms a hemostatic barrier on perivascular cells. Additionally, TF protects vital organs such as the brain, lungs, and heart from hemostatic damage. Under pathological conditions, in addition to triggering arterial thrombosis, TF can trigger venous thrombosis [69,70]. Free TF was also detected in blood in the form of microparticles, the main source of which is considered to be monocytes, endothelial cells, and platelets [71]. 

NFkB signaling induces an increase in the expression of TF, as TF has a binding site for NFkB [70]. Inhibition of NF-kB translocation by magnesium supplementation decreases the inflammatory response by lowering cytokine production and the expression of TF and prevents the occurrence of a proinflammatory and prothrombotic status. Conversely, in conditions of magnesium deficiency, inflammatory cytokines increase, and endothelial dysfunction sets in. This stimulates TF, which initiates a coagulation cascade, potentially leading to disseminated intravascular coagulation (DIC) [72]. 

In vitro studies have demonstrated that magnesium inhibits coagulation factors (prothrombin, thrombin, V, VII, and IX). Studies have shown that plasma prothrombin and antithrombin times are significantly shorter in magnesium-deficient animals than in animals without deficiency. Oral magnesium supplementation reduced the hypercoagulability state caused by a high-fat diet, i.e., a thrombogenic diet, while the use of magnesium sulfate on artificially induced intimate lesions suppressed platelet aggregation and thrombus formation [73,74]. Factor X activation by TF-FVIIa could be accelerated by magnesium and manganese ions independently of factor IX [75]. 

The antithrombotic effects of magnesium were observed in a canine model with percutaneous coronary intervention [76]. The intravenous administration of magnesium in a rat model of chemically induced carotid thrombosis showed that the antithrombotic action of magnesium largely depended on the time of administration. In order to be effective, it must be given before the formation of the thrombus [77]. 

Magnesium infusions in healthy volunteers revealed reduced platelet activity and increased bleeding time, without changes in fibrinolytic activity. An antiplatelet effect may be partially responsible for the beneficial effect of magnesium described in patients with acute myocardial infarction and preeclampsia [75]. Magnesium plays an important role in normal clot formation and lysis, while a decrease in the plasma magnesium concentration in patients with type-1 diabetes could generate thrombotic complications [78]. Magnesium sulfate in patients with end-stage liver disease and hypocoagulability was shown to improve clotting time [79]. 

A recent study conducted on patients with pregnancy hypertension who received treatment with magnesium sulfate alone or combined with nifedipine tablets showed significant increases in prothrombin time (PT), thrombin time (TT), and activated thrombin original time (APTT). In addition, a significant decrease in fibrinogen (Fib) was seen in all patients. In the combined treatment group, PT, TT, and APTT were higher and Fib was lower compared to the group treated only with magnesium sulfate [80]. 

Despite the physiological role of magnesium in coagulation being accepted, certain studies do not report clear results in this regard. Administration of magnesium sulfate in patients with preeclampsia did not influence the coagulation mechanism [81]. Similar results were observed in patients with AF who were undergoing mitral valve annuloplasty [82]. The antithrombotic effect of magnesium may be largely due to the inhibition of platelets, although anticoagulant or fibrinolysis-increasing effects were described [83]. 

The aim of this review is to summarize the available data regarding the role of magnesium in COVID-19 patients and its particularities in different clinical settings. 

## 2. Magnesium and COVID-19

Since the onset of the COVID-19 pandemic, numerous studies have investigated the impact of magnesium cations on the transmission and severity of COVID-19, as well as on patient outcome.

**Figure 1:**
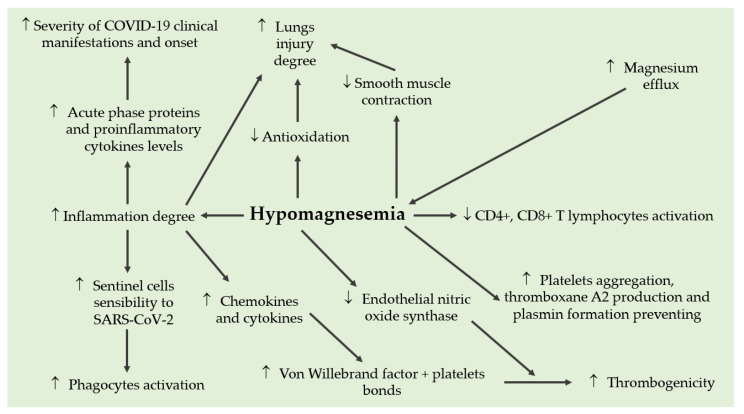
Pathophysiological role of magnesium deficiency in infection with SARS-CoV-2.

Magnesium deficiency could play a role in the pathophysiology of infection with SARS-CoV-2 (Figure 1). The primary manifestation of COVID-19 remains respiratory, but the virus can spread to other organs and tissues, complicating the clinical picture, culminating in multi-organ failure [72,84]. The key mechanisms involved in the disease include direct viral cytotoxicity, endothelial dysfunction, and exaggerated release of inflammatory cytokines [72,84,85]. 

Vascular endothelial damage is characteristic of COVID-19 and is likely associated with critical illness and death [72,84]. Magnesium deficiency increases oxidative stress and the release of proinflammatory cytokines from monocytes, macrophages, and leukocytes [32,55,72]. The administration of magnesium reduces these phenomena, which may be due to a reduction in the activation of nuclear factor NF-KB [55,72]. At the level of the pulmonary alveoli, magnesium deficiency increases tissue susceptibility to oxidative stress and decreases antioxidant defense, increasing damage and the possibility of a “cytokine storm” [52]. Moreover, by increasing proinflammatory cytokines, magnesium deficiency leads to endothelial dysfunction [28]. Thus, in the human body, magnesium deficiency can increase the risk of an inflammatory “cytokine storm” and can increase the possibility of damage to the vascular endothelium and a coagulation cascade, which is followed by disseminated intravascular coagulation phenomena [28,44,52,72]. 

It has been reported [72,78,86] that low serum magnesium levels are associated with increased thrombotic risk and delayed fibrinolysis, while low intracellular magnesium promotes the process of platelet-dependent thrombosis [45]. In vivo, Mg^2+^ has antithrombotic effects via reducing platelet aggregation and prolonging the blood coagulation time [77,87]. Moreover, it has been shown that magnesium has antithrombotic effects and reduces mortality in experimentally induced pulmonary thromboembolism [72,88]. All these effects suggest that, in patients with COVID-19, magnesium deficiency increases the risk of disseminated intravascular coagulopathy. 

Low magnesium induces a proinflammatory, prothrombotic phenotype in endothelial cells and promotes platelet aggregation, as well as beta-thromboglobulin and thromboxane release, resulting in the development of thromboembolism. Endothelial dysfunction and procoagulant status may explain the high incidence of thromboembolic events in patients with COVID-19 [72,84,85]. Magnesium deficits exacerbate the inflammatory response induced by SARS-CoV-2 and maintain and propagate the so-called cytokine storm, followed by acute respiratory distress syndrome, which favors the development of endothelial lesions and coagulopathy, the consequence of which is multiple organ dysfunction syndrome [84,85,89]. In addition, other symptoms that have been reported by patients with COVID-19, such as asthenia, myalgias, anxiety, depression, and insomnia, may be related to the presence of a Mg^2+^ deficiency [84,90,91]. 

A sufficient intracellular concentration of magnesium is necessary for the cytotoxic activity of T lymphocytes and natural killer (NK) cells [84]. Thus, an optimal level of magnesium plays a role in protecting cells against viral infections. In addition, people with magnesium deficiencies have a depressed immune response, an immune deficiency that is partially or almost completely corrected when magnesium supplements are administered [92,93]. Furthermore, intracellular free magnesium levels in natural killer cells and CD8 killer T-cells regulate their cytotoxicity [72,85]. 

Magnesium is required for the activation of vitamin D [72]. Magnesium deficiency can also reduce the level of active vitamin D (1,25 dihydroxyvitamin D) and affect the response of the parathyroid hormone [94]. This action can lead to “Mg^2+^-dependent vitamin D-resistant rachitis” [95]. Magnesium is also needed to inactivate vitamin D when levels become too high [96]. 

Thus, an optimal level of magnesium is necessary to ensure an optimal homeostasis for vitamin D. Vitamin D insufficiency is very common in patients with severe forms of COVID-19 [97]. This provides an important scientifical argument for vitamin D supplementation in patients diagnosed with COVID-19 infection. In patients who had their vitamin D levels measured one year before testing for COVID-19, the relative risk of becoming positive for COVID-19 was 1.77 times greater for those with a vitamin D deficiencies as compared to those who had sufficient levels [72,97]. 

Independently, both magnesium and vitamin D are important for the immune system. Together, these may be beneficial in COVID-19 infection, as magnesium is required for vitamin D activation. Given that magnesium and vitamin D are important for immune function and cellular resistance, a deficiency in either may contribute to the “cytokine storm” in SARS-CoV-2 infection [97,98].

In the cross-sectional study of ISARIC/WHO CCP-UK (COVID-19) [99], vitamin D deficiency or insufficiency was found in the majority of patients hospitalized with COVID-19 or influenza A and was associated with disease severity. Kalichuran et al. [100] also observed a high prevalence (82%) of vitamin D deficiency or insufficiency among hospitalized patients with COVID-19 and an increased risk of symptomatic disease in patients with vitamin D deficiency.

The SHADE study [101] revealed that a higher proportion of vitamin D-deficient individuals with SARS-CoV-2 infection became SARS-CoV-2 RNA negative, with a significant decrease in the inflammatory marker (fibrinogen) on short-term, high-dose cholecalciferol supplements. Furthermore, it has been observed that concomitant supplementation with vitamin D, vitamin B12, and Mg^2+^ in patients with COVID-19 may decrease the incidence of intensive care hospitalization and required oxygen therapy [89,102].

### 2.1. Interactions between Magnesium and COVID-19 Pathogenesis 

It is hypothesized that the initial stages of infection with SARS-CoV-2 could be dependent on cation Mg^2+^. The receptor for the viral protein spike S in the structure of SARS-CoV-2 is angiotensin-converting enzyme 2 (ACE2), which is present in many tissues, thus explaining the pulmonary and extrapulmonary manifestations of COVID-19 [103]. 

Similar to other coronaviruses, SARS-CoV-2 needs proteolytic cleavage of the S protein to activate the endocytic pathway for virus entry into the cell. It has been shown that host cellular proteases, including transmembrane protease serine protease 2 (TMPRSS2), cathepsin L, and pre-protein convertase-furin, participate in the cleavage of protein S and activate the entry of SARS-CoV-2 into the cell [84,93]. Magnesium plays an important role in the inhibition of these proteins [84]. The preliminary results of a study carried out by Fan et al. [104] suggested that magnesium treatment increased methylation, thus preventing TMPRSS2 transcription from the promoter and, consequently, reducing the expression of this proteolytic enzyme. These findings, if confirmed, provide another mechanism for the role of Mg^2+^ intervention in the prevention of COVID-19 and the treatment of early and mild forms of COVID-19 by modifying the phenotypic expression of the TMPRSS2 gene [84,104]. Furthermore, as a result of its antagonistic function with that of the calcium ion [84,92], magnesium could prevent the activity of furin, a calcium-dependent protein [92,105]. 

Taking into account what has been said before, it is clear that magnesium deficiency can promote the infectivity of SARS-CoV-2. Thus, in a large, retrospective cohort study [106] conducted in the United States (287,326,503 people from 1150 counties and 5,401,483 confirmed cases of COVID-19), it was shown that the average level of the cumulative incidence of COVID-19 in counties in areas with low magnesium content was significantly higher compared to control areas. At the same time, a significant negative nonlinear association was observed between magnesium concentration in the environment and the county-level cumulative incidence of COVID-19.

Once the virus enters the cellular level, magnesium deficiency can exacerbate the inflammatory response, contributing to the so-called cytokine storm, which has been shown to be involved in the pathogenesis of severe clinical manifestations of COVID-19 [84,85,107]. Moreover, magnesium deficiency mediators are associated with increased proinflammatory mediator concentrations [85,86,89]. Low magnesium serum levels, which often go undiagnosed, potentiate the reactivity to various assaults on the immune system and, therefore, become involved in the pathophysiology of multiple-organ damage from COVID-19. 

Low dietary intake of the micronutrient Mg^2+^ is associated with a higher incidence of diabetes and cardiovascular disease [89,108,109], medical conditions that are linked to severe complications in patients with confirmed SARS-CoV-2 infection [110,111]. 

Since it has been shown in experimental studies [112,113] that magnesium deficiency can act as a trigger of the inflammatory process, it was hypothesized that a magnesium-rich diet could lead to potential clinical benefits in COVID-19.

Thus, it has been observed [89,114] that a correct daily intake of fruits, vegetables, and whole grains significantly decreases the levels of some proinflammatory markers, such as lipopolysaccharide-binding protein, TNF-alpha, and IL-6. Veronese et al. [112] in a systematic review and meta-analysis showed the beneficial effects of Mg^2+^ supplementation in the significant reduction in different inflammatory markers (especially CRP) and increasing NO levels.

Considering the important role of magnesium in the regulation of inflammation, the immune system, and the coagulation cascade, we can hypothesize that an optimal level of magnesium may contribute to better prognoses for patients infected with SARS-CoV-2. This fact has also been noted in recent research [89,115,116,117,118,119,120], which suggests that magnesium has protective effects against the symptoms of COVID-19.

Furthermore, there have been studies showing that patients with hypomagnesemia are most frequently hospitalized [89,115,117,119,120,121], and there have also been reports refs.~[57,116,120,122,123] of lower magnesium levels in severe cases of COVID-19 compared to less severe cases. 

Thus, magnesium supplementation could have positive effects on many pathologies associated with COVID-19 in a similar way to some of the other therapeutic uses of Mg^2+^ in cardiovascular, nervous, coagulation disorders, and chronic inflammation.

On the other hand, hypermagnesemia was found in critical forms of COVID-19 [115,120]. This fact must be taken into account because hypermagnesemia can have adverse cardiovascular, neurological, and respiratory effects, which can worsen the evolution of a patient infected with SARS-CoV-2. In clinical practice, hypermagnesemia is usually caused by errors in the administration of magnesium-containing preparations [115,124]. In COVID-19, hypermagnesemia can also be explained by the rapid mobilization of the Mg^2+^ cation from tissues in conditions of stress or sepsis to necrotic events and does not seem to be related to kidney damage (nephropathy, on the contrary, being related to a decrease in Mg^2+^ concentration) [84,115,117,120].

### 2.2. Magnesium and Respiratory Damage 

As an enzyme activator, magnesium is essential for various physiological functions, such as the cell cycle, regulation of the metabolism, muscle contraction, and the maintenance of vascular tone. In SARS-CoV-2 infection, by inhibiting inflammation, oxidative stress, and smooth muscle contraction, magnesium intake can relieve pulmonary symptoms, protect the nervous system, and ameliorate cardiovascular function, liver and kidney damage, and blood glucose levels [85]. 

Thus, in the pulmonary tissue, magnesium sulfate inhibits proinflammatory molecules, including chemokines (macrophage inflammatory protein-2), cytokines (IL-6), prostaglandin E2, and cyclooxygenase-2, probably by inhibiting L-type calcium channels [107]. In an acute lung injury experimental model, magnesium sulfate ameliorated hydrochloric-acid-induced pulmonary histopathological lesions, including peribronchial inflammatory cell infiltration, alveolar septal infiltration, alveolar edema, and alveolar exudation [125]. Moreover, in experimental studies performed on mice, the administration of magnesium has significantly attenuated oxidative stress and the inflammatory response in lipopolysaccharide-induced acute lung lesions [85,126]. 

Furthermore, magnesium sulfate also inhibits the contraction of airway smooth muscles by blocking voltage-dependent calcium channels, which is a mechanism by which magnesium can be used in the treatment of bronchial asthma [85]. 

In severe forms of COVID-19, there is a strong inflammatory response and a “cytokine storm” following the increased release of proinflammatory cytokines (IFN-γ, TNF-α, interleukins, and chemokines), which can increase organ damage and accelerate the deterioration of a patient’s condition [127,128]. As mentioned before, magnesium has antioxidant and anti-inflammatory effects on the lungs, i.e., it decreases the inflammatory response, oxidative stress, and pulmonary inflammation, possibly by inhibiting IL-6, NF-κB, and L-type calcium channels [85,125,129]. Therefore, the administration of magnesium sulfate has good prospects for applications in the management of pulmonary symptoms from COVID-19 [85,118,125,129].

Infection with SARS-CoV-2 induces a prothrombotic and proinflammatory state that can increase the risk of serious thrombotic disorders in patients both during acute infection and in the post-COVID-19 period [130,131,132]. In COVID-19, aberrant inflammatory response in combination with hypoxia has been associated with endothelial dysfunction, imbalance of pro- and anticoagulant factors, and thrombin generation, leading to thromboembolic events [130,131,133]. In patients with COVID-19, the impairment of lung function can be linked to increases in blood pressure [133]. Moreover, pulmonary and venous thromboembolisms can cause severe cardiovascular problems, such as myocardial damage and cardiac and cerebral ischemia [130,131,132,133,134].

### 2.3. Magnesium and Cardiovascular Damage 

Coronary artery disease and hypertension are common coexisting disorders in patients with COVID-19 [85,135]. The affinity of the SAR-CoV-2 virus for the ACE-2 receptor has been proposed to be the core of the pathophysiology of the disease. Although SARS-CoV-2 infection predominantly affects the respiratory system, it can also cause cardiovascular complications, such as acute coronary syndrome, myocarditis, heart failure, and arrhythmias. Furthermore, COVID-19 can lead to severe ventricular dysfunction, even without signs and symptoms of pneumonia [136]. Several mechanisms have been implicated, such as an excessive inflammatory response to the primary infection, immunothrombosis, and myocardial damage. Patients infected with SARS-CoV-2 with a history of cardiovascular disease have increased mortality. Supplemental magnesium therapy can lower blood pressure, reduce the risk of atrial fibrillation, improve subclinical atherosclerosis, and prevent various other cardiovascular diseases [137,138].

### 2.4. Magnesium–Pregnancy Relationship 

In COVID-19 infection, pregnant women are at increased risk of more severe clinical symptoms and complications, especially in the respiratory system, due to associated high metabolic and oxygen consumption. Moreover, newborns from mothers who have had SARS-CoV-2 infection during pregnancy are more prone to complications such as fetal distress, premature birth, respiratory distress, and thrombocytopenia [85,139]. 

Magnesium sulfate is a category B drug according to FDA classification and, therefore, is safe and nonteratogenic [85]. In medical practice, MgSO_4_ is a drug that is frequently used in obstetrics for the effective prevention and control of premature labor, gestational hypertension, preeclampsia, and eclampsia with few side effects [139,140]. In addition, magnesium sulfate is used for its neuroprotective effect on the fetus because, in premature birth, administration to a pregnant woman before anticipated premature birth decreases the risk of cerebral palsy in premature infants, possibly through anti-inflammatory action [141,142,143,144]. Considering the beneficial effects of magnesium sulfate on pregnancy-induced hypertension, preeclampsia, and eclampsia and the neuroprotective effect on the fetus, timely administration of magnesium sulfate is strongly recommended for pregnant women infected with SARS-CoV-2 [85,142]. 

As described above, magnesium is involved in essential enzymatic reactions in cells, including the immune response [89]. Magnesium may contribute to the body’s immune response during and after SARS-CoV-2 infection by acting as a cofactor for the production of immunoglobulins and other processes required for T- and B-cell activity [85,89,145]. Citu I.M. et al. reported that pregnant women who, during pregnancy, received a diet supplemented with calcium, zinc, and magnesium, or with magnesium alone, did not have a different clinical course of disease during SARS-CoV-2 infection but showed a significantly higher titer of anti-SARS-CoV-2 antibodies. A low concentration of Mg^2+^ in the serum or a lack of supplementation with these micronutrients during pregnancy can lead to a less efficient immune response [145]. Although the observed results may suggest supplementing the nutritional intake of pregnant women with calcium, magnesium, and zinc, causality has not yet been determined.

## 3. Magnesium and “Long COVID-19” Syndrome 

As we have shown, magnesium is important in maintaining normal physiology, as it is involved in the regulation of inflammatory processes at the level of the blood vessels, pulmonary alveoli, and in coagulation and maintaining vascular tone [84,85,125]. As a result, magnesium homeostasis may influence susceptibility and response to SARS-CoV-2. 

Current studies have shown the existence of persistent and prolonged symptoms after acute infection with SARS-CoV-2, symptoms that have been included in the so-called long COVID-19 syndrome [85,90,91]. 

According to epidemiological studies [91,146], a large percentage of survivors of COVID-19 (ranging from 10% for those with documented infection to 50–80% for those hospitalized) have at least one symptom 3 months after confirmed SARS-CoV-2 infection or likely infection that cannot be explained by an alternative diagnosis. Common symptoms include fatigue, shortness of breath, cognitive dysfunction, and others and generally lead to a decrease in quality of life [91,147]. This condition is also recognized by different names, including long COVID, long-hauler syndrome, post-COVID syndrome, post-COVID-19 multisystem inflammatory syndrome, or post-acute COVID-19 syndrome. Long COVID-19 is a current concern for health professionals and requires multidisciplinary management [91,148]. 

It has been shown that patients with COVID-19 can experience a wide range of post-COVID-19 (long-term COVID-19) sequelae, including cardiovascular complications. COVID-19 can overstimulate the sympathetic system and induce an inflammatory cytokine storm and hypercoagulopathy state. These mechanisms may induce irreversible damage to the cardiovascular or respiratory system even after recovery from COVID-19, followed by irreversible complications, such as heart failure or decreased lung function. All these can increase the incidence of cardiovascular or cerebrovascular diseases among survivors of COVID-19. Currently, data on the actual incidence and relative risk of cardiovascular disease (CVD) after infection with COVID-19 are limited [128,149,150]. 

The simultaneous appearance of chronic fatigue, dyspnea, pain, and cough suggests the possibility of damage to the central nervous system [128,147]. SARS-CoV-2 has a neuroinvasive capacity, and sensory neurons appear to be the entry point into the central nervous system (CNS). In addition, the neuroinflammation caused by the cytokine storm can affect different regions of the brain. 

In this context, it is worth noting that, in the brain, Mg^2+^ influences biochemical processes involved in cognitive functions and in the integrity and stability of the cell membrane. It also exerts an antagonistic action against calcium and fights neuroinflammation [151,152]. Magnesium deficiency usually causes psychiatric symptoms, such as anxiety, insomnia, hyperemotionality, depression, headache, dizziness, and tremors, symptoms that can be found in post-acute COVID-19 syndrome. Furthermore, it has been suggested that magnesium deficiencies cause muscle weakness and myalgia. Magnesium is essential for all enzymes that use or synthesize muscle ATP and, therefore, for muscle energy production. Moreover, it regulates contraction and relaxation. In addition, magnesium contributes to the regeneration process of skeletal muscle fibers [85,151,153]. 

In conclusion, magnesium could be involved in the development of long COVID-19 syndrome and may aggravate symptoms or preexisting conditions. Therefore, the evaluation and, if necessary, correction of magnesium is essential to achieve complete recovery in patients with long COVID-19 syndrome.

## 4. Future Perspectives 

The COVID-19 pandemic is far from over, and recent findings have shown that COVID-19 could become a long-lasting disease that causes seasonal epidemics. Consequently, further research is needed on health risk factors associated with COVID-19 and people at risk of developing complications that can prevent or control the spread of COVID-19.

## 5. Conclusions

Magnesium acts as a cofactor of numerous enzymes, regulating ion channels and energy generation, and it is essential for maintaining normal cellular physiology and metabolism. The synergy between SARS-CoV-2 and ACE-2 receptors (signaled in pneumocytes and endothelial cells) via the S-spike protein transforms the ACE-2 pathways, causing acute injuries in the lungs, heart, and endothelial cells. Magnesium deficiency could play a role in the pathophysiology of SARS-CoV-2 infection. In the evolution of COVID-19, magnesium deficiency can increase the risk of triggering an inflammatory “cytokine storm”, damage to the vascular endothelium, and a coagulation cascade, which is followed by disseminated intravascular coagulation phenomena. Magnesium could be involved in the development of long COVID-19 syndrome and may aggravate symptoms or preexisting conditions.

## Data Availability

Not applicable.
